# Updated mechanisms of MASLD pathogenesis

**DOI:** 10.1186/s12944-024-02108-x

**Published:** 2024-04-22

**Authors:** Yuxuan Li, Peipei Yang, Jialu Ye, Qiyuan Xu, Jiaqi Wu, Yidong Wang

**Affiliations:** 1 Department of Cardiology, State Key Laboratory of Transvascular Implantation Devices, The Second Affiliated Hospital, Zhejiang University School of Medicine, Hangzhou, China; 2 Translational Medicine Center, The Affiliated Hospital of Hangzhou Normal University, Hangzhou, China; 3 Wenzhou Medical University, Wenzhou, China; 4 Department of Gastroenterology, The Affiliated Hospital of Hangzhou Normal University, Hangzhou, China

**Keywords:** MASLD, Lipid metabolism, Lipotoxicity, Therapeutics

## Abstract

Metabolic dysfunction-associated steatotic liver disease (MASLD) has garnered considerable attention globally. Changing lifestyles, over-nutrition, and physical inactivity have promoted its development. MASLD is typically accompanied by obesity and is strongly linked to metabolic syndromes. Given that MASLD prevalence is on the rise, there is an urgent need to elucidate its pathogenesis. Hepatic lipid accumulation generally triggers lipotoxicity and induces MASLD or progress to metabolic dysfunction-associated steatohepatitis (MASH) by mediating endoplasmic reticulum stress, oxidative stress, organelle dysfunction, and ferroptosis. Recently, significant attention has been directed towards exploring the role of gut microbial dysbiosis in the development of MASLD, offering a novel therapeutic target for MASLD. Considering that there are no recognized pharmacological therapies due to the diversity of mechanisms involved in MASLD and the difficulty associated with undertaking clinical trials, potential targets in MASLD remain elusive. Thus, this article aimed to summarize and evaluate the prominent roles of lipotoxicity, ferroptosis, and gut microbes in the development of MASLD and the mechanisms underlying their effects. Furthermore, existing advances and challenges in the treatment of MASLD were outlined.

## Introduction

Nonalcoholic fatty liver disease (NAFLD) is a metabolic liver injury characterized by excessive deposition of triglycerides (TG) in hepatocytes [1, 2]. It is an umbrella term for a range of liver diseases, including non-alcoholic fatty liver (NAFL), simple fatty liver (SFL), non-alcoholic steatohepatitis (NASH), cirrhosis, and hepatocellular carcinoma (HCC) in severe circumstances [2]. With the age of onset of NAFLD progressively decreasing and its relationship with cancer risk, research on NAFLD has become even more urgent [3].

The two-hit hypothesis was initially proposed to explain the pathogenesis of NAFLD, implying that a second strike of other causative factors needs to be involved in the development from simple hepatic steatosis to NASH [4, 5]. Nevertheless, this hypothesis is gradually being revised as new theories emerge. Indeed, various factors, including genetics, dietary habits, air pollution, alcohol, smoking, and insulin resistance, influence the development of NAFLD [6–8]. Notably, oral health, such as periodontitis, has been reported to be linked to the development of NAFLD [9–11]. With an evolving understanding of the pathogenesis of NAFLD, its terminology has shifted to MASLD, whilst that of NASH was redefined as metabolic dysfunction-associated steatohepatitis (MASH) [12]. Given the correlation between MASLD and metabolic syndrome, it often coexists with metabolic disorders [13]. At the same time, MASLD involves multiple pathogenic molecular pathways, thus leading to heterogeneity in its pathogenesis and clinical manifestations [14–16]. Based on these findings, a new theory is proposed referring to conditions beyond normal hepatic metabolic capacity, excessive deposition of toxic lipids, hepatocellular stress, injury, and death [17]. The intricate molecular mechanisms underlying MASLD are gradually elucidated. For instance, transmembrane 6 superfamily member 2 (TM6SF2) plays a key role in MASLD, and participates in the regulation of lipid metabolic processes [18, 19]. Nevertheless, there is still no clinically recognized agent for the treatment of MASLD. Therefore, there is an urgent need for further exploration of its pathogenesis. Herein, we aim to conclude molecular mechanisms of MASLD, thus laying a theoretical reference for subsequent diagnostic and therapeutic development.

## The pathogenesis of MASLD

**Fig. 1:**
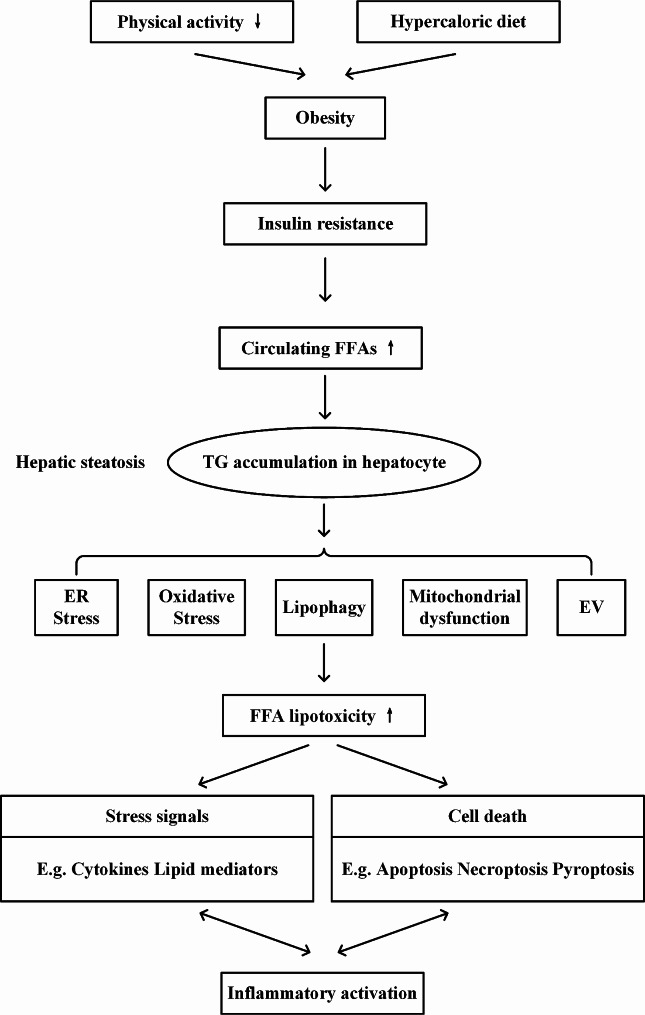
Potential sources and mechanisms of hepatic fat accumulation. Genetic risk, lifestyle, and metabolic factors all contribute to hepatic steatosis. Lipid accumulation in hepatocytes leads to lipotoxicity, which activates oxidative stress-related molecules and signals and transmits them between cells in the form of extracellular vesicles or diffusion, thereby triggering the cell death program and pushing hepatic steatosis toward inflammation and fibrosis. FFA, free fatty acid; TG, triglyceride; ER, endoplasmic reticulum; EV, extracellular vesicles

Risk factors for MASLD include obesity, insulin resistance, hypertension, and hypertriglyceridemia (Fig. 1). As anticipated, the global prevalence of MASLD increases in parallel with global obesity rates. A meta-analysis documented a prevalence of MASLD of 75.27% in the obese population [20]. Disorders of lipid metabolism are the primary cause of fatty liver. MASLD is predominantly hallmarked by triglyceride accumulation in hepatocytes. Earlier studies have established that a reduction in the level of triglyceride lipase could effectively lower the risk of high-fat diet-induced MASH in mice [21]. While the majority of MASLD patients are obese adults, its incidence in leaner individuals is on the rise, leading to its designation as “lean liver disease” [22].

Hepatic steatosis can induce oxidative stress, organelle dysfunction, apoptosis, and other pathophysiological changes, thereby promoting the progression of MASLD to MASH and liver fibrosis [23]. Steatosis is the result of caloric overload and the accumulation of large amounts of triglycerides in hepatocytes. The metabolism of free fatty acids (FFA), substrates for TG formation, is of particular importance in this process. The hepatic pathway for fatty acid acquisition encompasses exogenous dietary intake, as well as de novo lipogenesis (DNL). Numerous studies have described that the fatty acids (FAs) are tightly regulated in tissue despite fluctuations in FFA intake. Consequently, increasing FFA intake will not significantly affect its concentration in tissue, but prolonged intake exceeding 10% of total energy can have detrimental effects on health [24]. The close association between the liver and adipose tissue is related to the uptake and synthesis of lipids in the liver. FAs are released into the bloodstream from adipose tissue and transported to the liver bound to plasma proteins. Membrane-bound fatty acid transport proteins (FATPs), namely FATP2 and FATP5, which have been identified in the liver, facilitate the uptake of fatty acids and make them available for hepatic lipid metabolism [25]. According to earlier studies, deficiency of FATPs mitigated the accumulation of hepatic FAs in a high-fat model of mice [26], demonstrating the promising role of FATPs during hepatic steatosis.

The level of the fatty acid translocase CD36 is increased on the plasma membrane of hepatocytes of MASH patients, while inhibition of CD36 palmitoylation ameliorates intracellular lipid accumulation, providing a viable therapeutic strategy for the treatment of MASLD [27]. Moreover, the levels of two key enzymes in DNL, acetyl-CoA carboxylase (ACC) and fatty acid synthase (FAS), are elevated in MASLD patients, implying that DNL plays an essential role in lipid deposition. Although the mechanisms underlying the activation of DNL in MASLD remain to be elucidated, dysregulation of DNL is considered crucial for fatty liver formation. Increased fatty acid oxidation does not eliminate hepatic lipid deposits but induces dysfunctional mitochondria to produce excessive ROS, thereby contributing to oxidative stress and leading to the development of liver disease [28]. Elevated levels of triglycerides in circulation facilitate FFA infiltration into non-fat tissues, leading to abnormal accumulation of fat and dysfunction of non-fat cells. Recently, the concept of lipotoxicity was introduced. Lipotoxicity drives hepatocyte death by activating inflammatory pathways [29–31].

As previously mentioned, the precise mechanisms underlying MASLD remain to be elucidated. This article focused on organelle dysfunction related to hepatocyte injury in the progression of MASLD. The concept of lipotoxicity has been proposed as one of the driving factors leading to MASH, which causes dysregulation of hepatic lipid metabolism and, thus, hepatocellular damage. Enhanced lipotoxicity results in substantial hepatocyte damage via endoplasmic reticulum (ER) stress, activation of inflammatory vesicles, and cell death (Fig. 1) [18]. Meanwhile, ferroptosis and gut microbial dysbiosis have also emerged as research hotspots in recent years for exploring the underlying mechanisms of MASLD pathogenesis.

## Lipotoxicity in MASLD

### ER stress

As is documented, the unfolded protein response (UPR) refers to a defensive response that maintains the dynamic homeostasis of the endoplasmic reticulum environment, but its prolonged activation induces apoptotic pathways that ultimately cause hepatocyte damage and death [32, 33]. Under different stressful conditions, molecular chaperones undergo ectopic translocation, phosphorylating the three ER receptors and subsequently activating the immunoglobulin-regulated enhancer 1 (IRE1), protein kinase RNA-like endoplasmic reticulum kinase (PERK) or activating transcription factor 6 (ATF6) signaling pathways [34, 35]. Previous studies have reported that ATF6α, PERK/eukaryotic initiation factor-2α (eIF2α), and IRE1α/X-box binding protein 1(XBP1), three ER stress sensing pathways, can regulate the development of MASLD by altering lipid synthesis [36]. Similarly, all three pathways promote the synthesis of C/EBP-homologous protein (CHOP) and directly affect ER stress [37–39]. The down-regulated expression of GRP78, IRE1α, XBP1, phosphorylated eIF2α, CHOP, and ATF6 attenuates inflammation and HFD-induced ER stress. Salubrinal regulates lipid metabolism and down-regulates CHOP expression via the eIF2α signaling pathway to alleviate lipid accumulation in the liver, providing strategies to mitigate MASLD [40]. Narjes et al. demonstrated that the administration of empagliflozin for five weeks reduced hepatocyte apoptosis and relieved MASLD in HFD-fed mice [41]. Meanwhile, a newly discovered traditional Chinese medicine, Berberine, effectively inhibited PERK-ATF4-CHOP pathway in mouse hepatocytes and macrophages but did not affect ATF6, IRE1α, and GRP78 protein levels [42]. Valdecoxib (VAL), a COX-2 inhibitor, was reported to alleviate lipid accumulation in hepatocytes, and this protective effect was dependent on reduction of ER stress [43]. Therefore, modulating hepatic ER stress represents an effective approach for the treatment of MASLD.

**Fig. 2:**
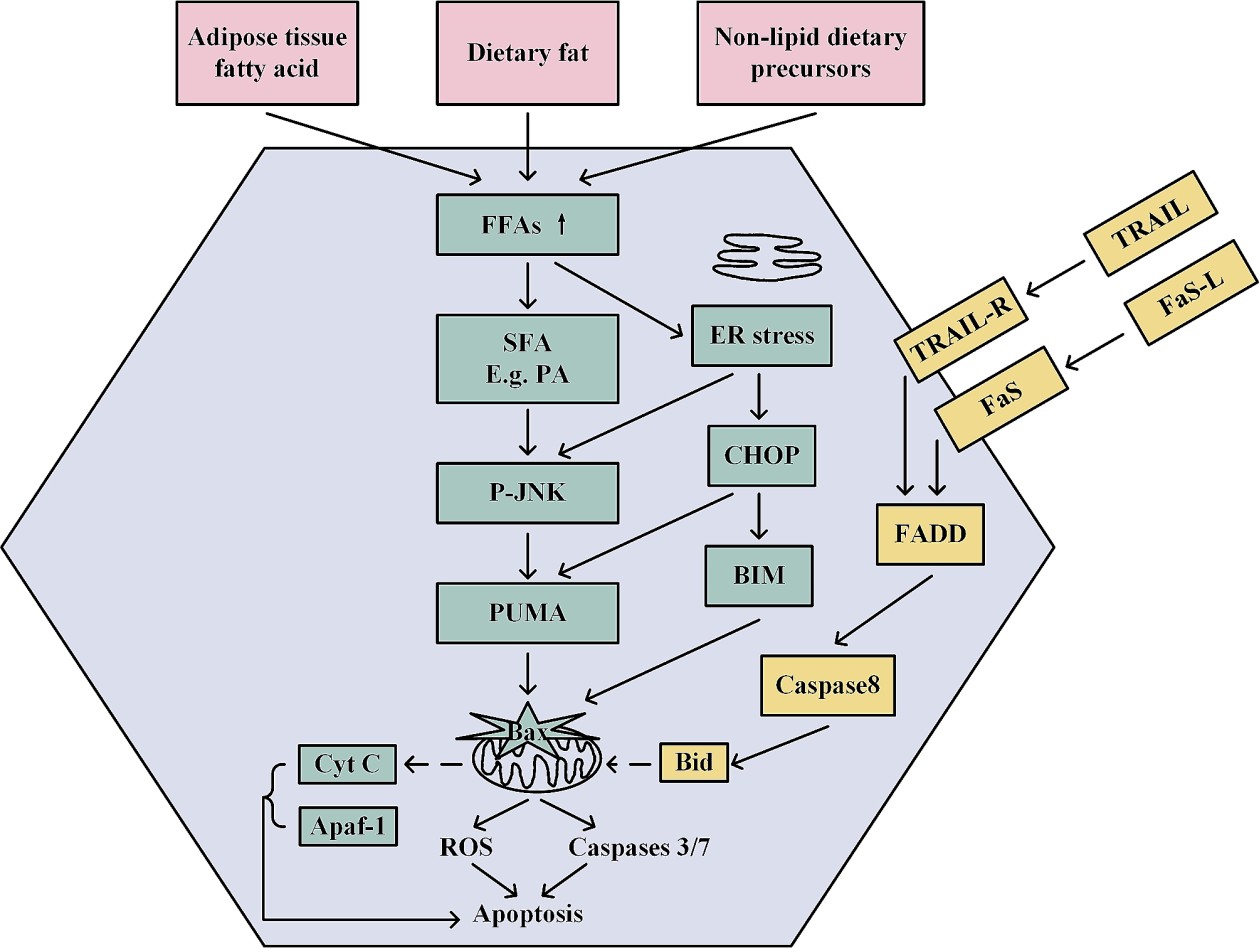
Molecular mechanisms associated with hepatocyte lipotoxicity and apoptosis. There are 3 major sources of fatty acids in the liver, including dietary intake, self-synthesis from scratch, and catabolism by peripheral adipose tissue. FFA is transported into the hepatocytes to synthesize triglycerides, which leads to hepatic steatosis. Lipotoxicity induces death receptor signaling pathways that recruit caspase 8 to cleave Bid and regulate apoptosis. Excess SFA accumulates in the ER and induces ER stress, which in turn induces the transcription factor CHOP and mediates the onset of JNK. CHOP not only interacts with activated c-jun to upregulate the transcription of the pro-apoptotic BH3 protein, PUMA but also increases the expression of another BH3-only protein, Bim, which synergistically activates the pro-apoptotic protein, Bax. Bim and PUMA synergistically activate the pro-apoptotic protein Bax, which causes mitochondrial dysfunction and induces apoptosis through the release of cyt C and the activation of caspases proteases. Mitochondrial dysfunction, on the other hand, also leads to the overproduction of reactive ROS, which causes oxidative stress and further induces cell death. FFA, free fatty acid; SFA, saturated fatty acid; ER, endoplasmic reticulum; oxygen species

Overall, ER stress remains a popular topic for exploring the pathogenesis of MASLD, wherein CHOP plays a critical role (Fig. 2). Moreover, molecules like XBP1 are also closely related to URP activation and constitute a potential novel target for suppressing ER stress. A growing body of evidence has acknowledged the important role of maladaptive UPR in the progression of liver disease [44, 45]. Therefore, future research may focus on liver-specific ER stress modulators. Additionally, studies with IRE1 and ATF6 in MASLD are warranted to determine the effects of IRE1 or ATF6 inhibitors on MASLD and to identify new targeted drugs for this scenario.

**Fig. 3:**
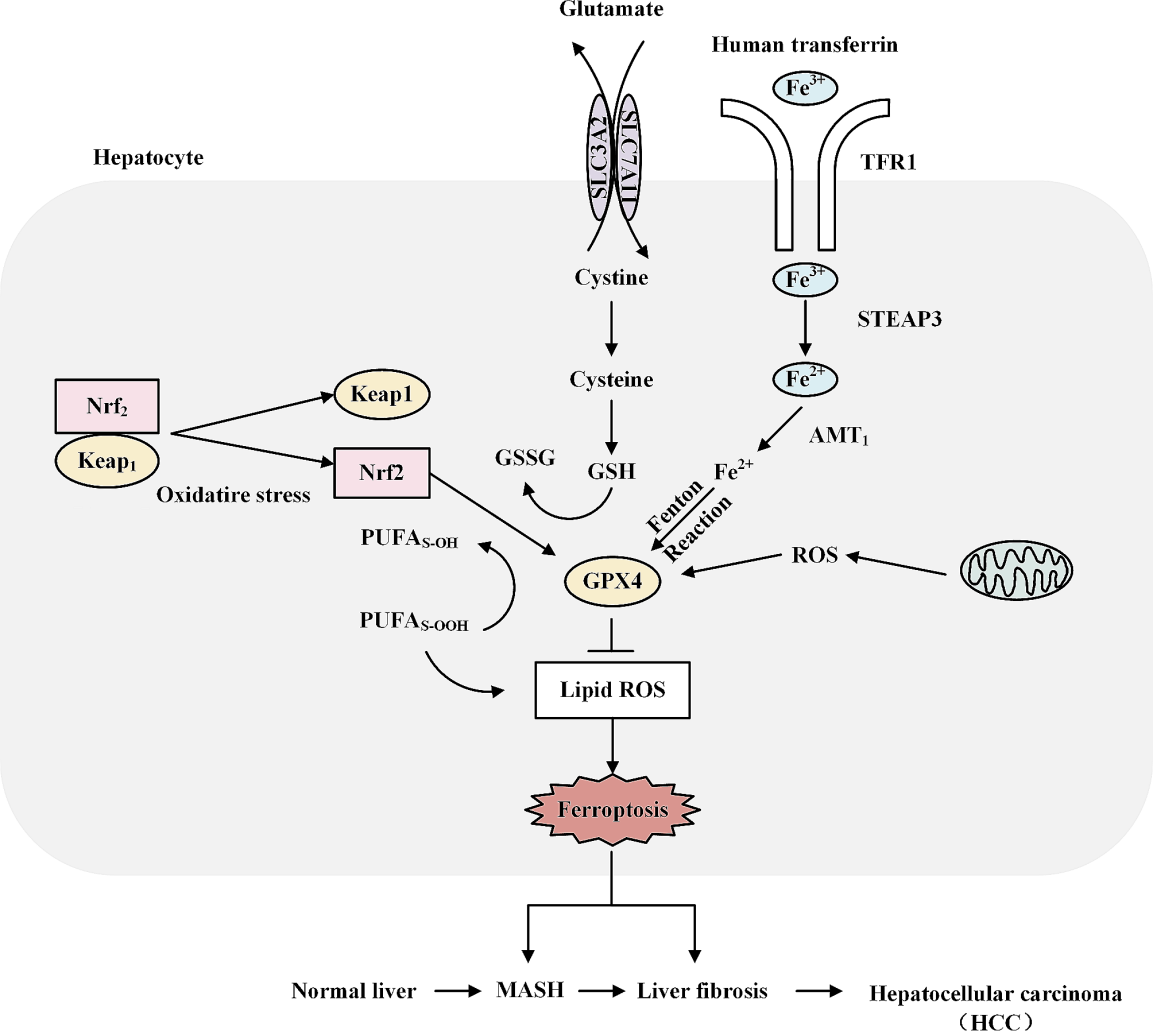
The regulatory mechanisms of ferroptosis in MASLD and its effects on the progression of MASLD. Cystine enters the cell via SLC7A11 and SLC3A2 embedded in the surface of the cell membrane and is then oxidized to cysteine, which is catalytically synthesized into GSH.GPX4 utilizes the ability of GSH to convert lipid peroxidation of L-OOH to L-OH, losing its peroxidative activity and thus protecting against the induction of ferroptosis. Nrf2 inhibits ferroptosis by regulating GPX4 and iron metabolism. PUFA binds to phosphatidylethanolamine(PE) to form polyunsaturated fatty acids phospholipids, the latter of which are susceptible to lipoxygenase(LOX)-mediated free radical-induced oxidation that induces ferroptosis. Fe3 + is uptaken by TFR1, reduced to Fe2 + by STEAP3, and later transported into the cytoplasmic unstable iron pool (LIP), a regulator of iron metabolism, and two proteins in the family of zinc-iron-modulated proteins (ZIP8/14), however, in the presence of excess Fe2+, can induce cellular ferroptosis by increasing reactive oxygen species generation and promoting lipid peroxidation formation via the fenton pathway. GSH, glutathione; GPX4, glutathione peroxidase 4; Nrf2, nuclear erythroid-related factor 2; PUMA, P53-up-regulated modulator of apoptosis; TFR1, transferrin receptor 1 P53-up-regulated modulator of apoptosis; TFR1, transferrin receptor 1

### B-cell lymphoma-2(Bcl-2) family proteins and apoptosis

Saturated fatty acids induce apoptosis in hepatocytes (Fig. 2) through the up-regulation of Bcl2-associated x (Bax) and Bcl-2 interacting mediator (Bim) of cell death in the c-Jun N-terminal kinase (JNK)-a dependent pathway and upregulation of the BH3-only protein (bcl-2 homolog3r domain only proteins). P53-up-regulated modulator of apoptosis (PUMA) has also been detected in human samples and animal models with MASH [46–48]. Upregulation of hepatic PUMA expression is governed by JNK1 and CHOP transcription [49, 50]. The pro-apoptotic protein PUMA interacts with Bax to mediate mitochondrial apoptosis and mobilize caspase activity [51], which is strongly associated with the progression of MASLD and MASH [52]. In addition, Bim likewise contributes to adipocyte apoptosis and interacts with PUMA to mediate palmitate-induced hepatocytotoxicity [53, 54]. Thus, the deficiency of Bim and PUMA in hepatocytes might alleviate apoptosis and provide a promising direction for the development of MASLD. Prior experiments have demonstrated that the JNK inhibitor JM-2 attenuated adipocyte apoptosis and MASLD by limiting palmitic acid (PA)-induced liver fibrosis and lipid metabolism [55]. Tamoxifen was found to inhibit hepatic fat accumulation by mediating the JNK/MARK signaling pathway, suggesting potential implications for MASLD treatment [56]. At the same time, the protein expression levels of anti-apoptotic such as Mcl-1, which comprises four BH structural domains, were reduced in FFA-exposed hepatocytes [57]. Fernando J et al. revealed that FFA regulates Bim expression by activating FoxO3a and proposed a strategy for the potential treatment of MASH [54]. Cellular Keap1 protein responds to PA-induced hepatotoxicity by undergoing degradation via the p62-dependent autophagy pathway to activate Bim and PUMA, triggering apoptotis [58]. Activation of the pro-apoptotic protein Bax results in cell death by altering mitochondrial membrane permeability and stimulating the caspase response. Given that the level of cytokeratin fragments produced by caspase catabolism serves as a marker in the development of MASLD, caspase inhibitors are a prospective treatment direction [59]. Various caspase inhibitors (Q-VD-OPh, Belnacasan/VX765) have been pioneered, offering candidate therapeutic strategies for MASLD [60, 61]. An aldose reductase inhibitor, epalrestat, acts as the upstream of caspase-1 to inhibit NLRP3 inflammatory vesicles and ameliorate MASH pathology [62]. Recently, Elodie Bosc et al. presented a definitive perspective on caspase-2-specific inhibitors for the treatment of MASLD, while their studies exposed that 3-(S)-neopentyl proline (LJ3a) and 6-methyl-tetrahydro-isoquinoline (LJ2a) inhibited caspase-2-mediated activation of SREBP2 [63].

Taken together, hepatic apoptosis induced by caspases, Bcl-2 family proteins, and c-Jun N-terminal kinase has an impact on influences MASLD/MASH activation. Multiple apoptosis inhibitors have been identified as potential treatments for MASH. Several clinical trials targeting apoptosis in MASH patients are currently ongoing and are expected to be an attractive field for mitigating the progression of MASLD in the future.

### Mitochondrial disorder

Bedair Dewidar et al. constructed a MASLD mouse model and measured its mitochondrial respiration. The findings showed that hepatic mitochondrial respiration increased due to fatty acid oxidation (FAO)-driven adaptation to metabolic disorders in response to alterations in lipid metabolism, systemic oxidative stress, and other changes [64]. Interestingly, MASH patients, on the other hand, exhibit increased mitochondrial uncoupling and leakage activity, structural defects, and decreased function, leading to excessive oxidative stress in the liver [65, 66]. Voltage-dependent anion channel (VDAC) acts as an early sensor of lipotoxicity. In response to lipid accumulation, VDAC exhibits a reduction in GSK3-mediated phosphorylation, modulating the permeability of the outer mitochondrial membrane. This allows a large influx of water and calcium into the mitochondria, leading to organelle edema as well as the release of cyt C that eventually induces cell death [67, 68]. Furthermore, in PA-mediated lipotoxicity, the mitochondrial protein Sab interacts with JNK, leading to impairment of the mitochondrial electron respiratory chain, increased ROS production, and ultimately activation of apoptosis [69]. Mitochondrial autophagy eliminates ROS to maintain mitochondrial homeostasis and holds significant implications for the treatment of MASLD [70]. At the same time, excessive intake of SFAs affects the structure and function of hepatic mitochondria and limits respiratory efficiency, which induces liver damage [71]. Additionally, cAMP-response element binding protein H (CREBH), which specifically regulates hepatic lipid metabolism, has been implied as a therapeutic target for hepatocyte resistance to mitochondrial oxidative stress and inflammation in MASH [72].

Collectively, the previous study demonstrated that SFA plays a vital role in the progression of hepatic steatosis as well as MASLD by impacting the regulation of mitochondrial function [73, 74]. A large number of studies conducted in mice and humans have determined that mitochondrial respiration is typically increased in the early stages of hepatic steatosis [64, 75]. However, this role of mitochondrial respiration in bioenergetics remains underexplored, and there is growing evidence that reactive oxygen species and lipid peroxidation are generated in patients with hepatic steatosis, thereby impairing the respiratory chain and inducing apoptosis [76]. This culminates in a vicious cycle in which massive ROS production exacerbates apoptosis. Although a link between apoptosis stimulated by mitochondrial dysfunction and MASLD has been proposed [77, 78], further research is necessitated to determine the mechanisms by which SFA regulates mitochondrial respiration, electron leakage, and other pathways that may play a role in the initiation of apoptotic processes.

### Lysosomal dysfunction

Studies have uncovered that the relative absence of lysosomal acid lipase in the liver of patients with MASLD reflects lysosomal dysfunction [79, 80]. Of note, damaged autophagy in MASLD is caused by defective lysosomal acidification and lysosomal calcium retention [81, 82]. FFA contributes to the translocation of Bax to lysosomes in hepatocytes, rendering lysosomes unstable and releasing cathepsin B (ctsb) into the cytoplasm, thereby mediating lipotoxicity [83]. Autophagy refers to the mechanism responsible for removing dysfunctional intracellular components through lysosomal degradation and has been shown to mediate intracellular lipid metabolism, a phenomenon termed lipophagy [84]. Recently, Karla K. Frietze et al. unveiled that DDX58 deficiency impairs autophagy and that DDX58 overexpression prevents PA-induced death, indicating that autophagy is a new direction for the treatment of MASLD [85]. At the same time, Liu et al. concluded that autophagy inhibition could be regarded as a potential contributor to MASLD and highlighted the potential of Nrf2, an anti-oxidative stress transcription factor, for the treatment of fatty liver disease [86]. Previous studies have also reported that unsaturated fatty acids exert a protective effect on organelle dysfunction induced by saturated fatty acids [87]. Liu et al.‘s study further demonstrated that oleic acid (OA) supplementation attenuated lysosomal dysfunction caused by disrupted autophagy, presenting OA as a therapeutic agent for metabolic diseases, including MASLD [88]. Of note, cholesterol from dead hepatocytes induces macrophage lysosomal dysfunction, thereby promoting the occurrence of MASH [89].

### Ferroptosis in MASLD

Ferroptosis, first introduced by Stockwell’s work, is an iron-dependent type of non-apoptotic cell death. It is driven by the accumulation of lipid peroxides, unlike the universal forms of cell death (autophagy, apoptosis, and necrosis) [90]. Several studies have corroborated that ferroptosis exhibits unique morphological, biochemical, and genetic features [90–92]. In terms of cellular morphology, ferroptosis is characterized by smaller mitochondrial volume, increased membrane density, and a lower number of cristae, along with insignificant changes in the nucleus [93]. Biochemically, it is usually accompanied by increased lipid peroxidation, elevated ROS levels, and related genetic changes [94]. The regulation of ferroptosis is closely linked to a wide range of metabolisms [95]. Consequently, mounting evidence emphasizes the close relationship between ferroptosis and liver metabolic diseases [96, 97], including MASLD [98, 99]. Given the relevance of ferroptosis to MASLD, understanding and elucidating the exact pathogenesis of ferroptosis in affecting MASLD is significant. In other words, such insights may contribute to the development and application of potential therapeutic target drugs for MASLD in the future.

Considering the susceptibility of the liver to iron metabolism and lipid peroxidation, a substantial body of evidence supports the notion that ferroptosis may be closely associated with the pathogenesis of MASLD. Li et al. conducted RNA-seq analysis and revealed that ferroptosis was promoted by arachidonic acid metabolism in a methionine-choline deficient (MCD)-diet induced MASH mouse model, suggesting that ferroptosis may be a therapeutic target for MASH [98]. The role of ferroptosis as a trigger for eliciting inflammation in MASH was noted in a study carried out in 2019 [100]. Although the underlying mechanisms of ferroptosis in MASLD remain enigmatic, research on ferroptosis has remained vigorous in recent years. Tong et al. observed that the ferroptosis inhibitor liproxstatin-1 restricted hepatocyte apoptosis, pyroptosis, and necroptosis in a mouse model of MAFLD [101]. In addition, Liu et al. documented the antioxidative and anti-inflammatory effects of zeaxanthin (ZAF), an inhibitor of ferroptosis, in FFA-induced HepG2 cells [102]. Most ferroptosis inhibitors can alleviate liver damage and offer possible strategies for intervening in the development of MASLD and inflammation. MASH progression is regulated by numerous ferroptosis-related genes. Among them, GPX4 and Nrf2 stand out as protective mechanisms that inhibit ferroptosis (Fig. 3). Further study of these genes may be beneficial for MASLD treatment. GPX4 is an essential target in MASH, and up-regulation of GPX4 expression can effectively alleviate hepatic metabolic injury. However, a precise understanding of the genetic modulation of GPX4 is still lacking. Further elucidation of the role of GPX4 in MASH via depletion or overexpression in hepatocytes is required. Thymosin beta 4 (Tβ4) inhibits ferroptosis and optimizes lipid metabolism in HFD-induced MASLD mice by protecting against hepatocyte injury and upregulating GPX4 [103]. Zhang et al. identified inhibition of the tripartite motif-containing (TRIM59) family as a possible candidate for MASLD treatment, given that it promotes ferroptosis and lipoatrophy by enhancing GPX4 ubiquitination [104, 105]. Targeted induction of inducible-GPX4 (iGPX4) promotes deleterious ferroptosis under MAFLD conditions, contrary to the effect of canonical-GPX4 (cGPX4). Thus, the conversion of GPX4 isoforms provides new horizons for ferroptosis regulation [106]. Nevertheless, MCD dietary modeling is not a complete substitute for physiologically relevant diets in MASLD, posing limitations on data interpretation on GPX4 isozyme conversion.

Given the complex involvement of multiple metabolic pathways and ambiguity in the mechanisms of MASLD, the role of ferroptosis in the prevention and treatment of MASLD deserves further in-depth investigation. Liproxstatin-1 (LPT1), a ferroptosis inhibitor, significantly inhibits PANoptosis (a combination of apoptosis, pyroptosis, and necroptosis) in MASLD and thus attenuates hepatic lipoatrophy, which is conducive to the prevention and treatment of MASLD [101]. In summary, although ferroptosis inhibitors have shown promise in the treatment of MASLD, long-term safety, and optimal dosing regiments need to be further evaluated. In addition, prospective studies remain a prerequisite prior to performing clinical trials.

## Gut microbial dysbiosis in MASLD

Gut microbes (GM) play a pivotal role in metabolic homeostasis, and disruptions in gut microbial homeostasis can lead to the development of a variety of metabolic disorders, including MASLD [107, 108]. Dysbiosis of the intestinal flora may increase the permeability of the intestinal barrier, which leads to enhanced absorption of fatty acids [109]. On the other hand, intestinal damage may induce liver damage by exposing the liver to bacteria and their products, such as endotoxins [110]. GM can also chemically modify bile acids (BA) and affect glucose and lipid metabolism in the liver by modulating the BA signaling pathway, thereby influencing the pathogenesis of MASLD [111]. Observations from several studies indicate that the diversity of gut microflora is generally lower in MASLD patients than in the healthy population. Abundance of Gram-negative bacteria (e.g., Bacteroidetes) is enriched, while abundance of Prevotella is lower [112, 113]. Mbaye et al. identified a potential association between lactic acid bacteria and MASH by statistically analyzing the microflora of 10 MASH patient cases and controls [114]. It is universally recognized that gut microbial metabolites play a key role in regulating MASLD pathogenesis. Treatment using Lingguizhugan decoction (a traditional Chinese medicine prescription) for four weeks effectively ameliorated MCD-induced MASH features and reversed the alterations in the abundance of 57 gut microbial metabolites, suggesting the potential of gut flora and its metabolites as therapeutic MASH targets [115]. Short-chain fatty acids (SCFAs), as one of the most common products of carbohydrates (CHOs) and proteins, exerts an integral positive effect in MASLD. Furthermore, clinical evidence demonstrated that SCFA levels are significantly lower in patients with MASH [116]. For example, recent studies have evinced that commensal microbe-derived acetate moderates the development of MASLD through the free fatty acid receptor 2 (FFAR2) signaling pathway in mouse models [117]. In addition, ethanol, an essential gut microbial metabolite, is also strongly associated with MASLD [118]. The role of microbial-derived ethanol in the pathogenesis of MASLD warrants further investigation. By intervening with the selective alcohol dehydrogenase (ADH) inhibitor 4-methyl pyrazole in MASH patients and controls, Meijnikman et al. demonstrated that the elevated ethanol production in patients with MASLD may be driven by the gut microbiome [118]. Elevated levels of alcohol-producing bacteria in the gut and blood ethanol concentrations have been detected in MASH patients, compared to those with hepatic steatosis, indicating that endogenous alcohol could be implicated in the pathogenesis of MASLD [118–120]. The role of lipid factors in the complex mechanisms of MASLD cannot be overlooked, and recent discoveries suggest potential interactions with gut microbiota homeostasis, offering promising therapeutic directions for delaying MASLD progression [121]. Apelin (APLN) has been hypothesized to be associated with MASLD progression and the gut microbiota, particularly associated with the abundance of Enterobactericae, Prevotellaceae, and Lactobacillaceae. Therefore, changing Apelin levels by altering the gut microbiota may be an effective approach to preventing the progression of MASLD [122]. At present, modulation of gastrointestinal microbiota has been widely studied and applied as a novel target for the treatment of MASLD. Recent studies have found that alanyl-glutamine (AG), a dipeptide comprising alanine and glutamine, can alleviate HFD-induced MASLD by modulating gut microbial composition [123]. The recent discovery that Oat beta-glucan regulates the gut microbiota, and mitigates hepatic inflammation and fibrosis in MASLD, indicating a well-tolerated approach to preventing MASLD [124]. In addition, Liu et al. explored interplay of apolipoprotein H (APOH) and gut microbiota in MASLD [125]. They concluded that down-regulation of APOH induces gut microbial disorders and exacerbates fatty liver, highlighting the role of APOH in fatty liver [125]. Notably, oral health, such as periodontitis, has been theorized to be linked to the development of MASLD [126]. In addition, emerging evidence suggests a relationship between oral and gut microorganisms [127, 128]. The oral-gut-liver axis concept has been proposed, portraying periodontal treatment as a potential treatment and prevention strategy for MASLD [129]. Psoriasis, a prevalent dermatological disease affecting 125 million people worldwide, is closely related to liver disease due to systemic inflammation, as evidenced in various studies [130, 131]. Furthermore, beyond the chronic inflammatory status, intestinal dysbiosis exacerbates the pathogenesis of psoriasis-like phenotypes through fluctuations in fatty acid metabolism [132]. On the other hand, the microbiome plays a critical role in preserving the homeostasis of T cells and immunoglobulin A production in the gastrointestinal tract [133]. At present, the causal relationship between specific intestinal flora and MASLD has not been fully elucidated. Further exploration is required to determine the impact of regulating the intestinal flora and metabolites as a therapeutic strategy for MASLD in the future.

## Research prospects for therapeutic strategies

At present, no licensed pharmacological therapy has been approved for the treatment of MASLD, and drug development in MASLD trials remains challenging due to the high variability in histopathological assessment of liver biopsies [134]. Nevertheless, research into treatment options for MASLD is underway, and this review aims to summarize current treatment advances to lay a theoretical reference for future studies.

A healthy diet combined with regular exercise remains the first-line non-pharmacological intervention for the treatment of MASLD [135]. A meta-analysis conducted by Shirin Hassani Zadeh et al. determined that the Mediterranean dietary (MD) pattern reduced the risk of MASLD by 23% compared to the Western dietary pattern [136]. Therefore, lowering ketosis and free fatty acid intake and controlling carbohydrate consumption enhances hepatic metabolic function and reduces the risk of liver disease, thereby preventing the development of MASLD [137]. In addition, the ketogenic diet is involved in controlling MASLD by altering the functional state of liver mitochondria, promoting ketogenesis, and improving hepatic fat metabolism [138, 139]. However, the precise mechanisms of ketosis in MASLD remains unknown [138, 139]. Li et al. explored the therapeutic effects of diet in MASLD patients and determined that intermittent fasting (IF) was more beneficial in reducing the lipotoxicity of MASLD compared to conventional therapies than switching to a normal diet (ND) [140]. Recently, a high-fat/high-fructose diet including 10% of bean leaves has also been found to ameliorate lipoatrophy and lipid peroxidation in fatty liver disease, providing new perspectives for the prevention and treatment of MASLD and MASH [141]. Regular exercise may also alleviate glycolipid metabolism disorders and thus be effective against MASLD, but there is no clear evidence regarding the therapeutic effects of exercise modalities in patients with MASLD in the literature [142, 143].

Although weight loss, dietary modifications, and exercise are recommended for the prevention and treatment of MASLD, they are not a long-term solution, necessitating additional research on effective therapeutic agents for MASLD. The guidance in 2018 stated that omega-3 fatty acids may be effective in MASLD [144]. There have been several subsequent studies addressing the benefits of omega-3 fatty in the treatment of MASLD, among which Mohammad Mohammadi et al. identified the potential synergistic efficacy of fish oils (FOs) and chicory acid (CA) in reducing hepatic fat accumulation in a MASLD in vitro model [145]. Ascribed to the unclear pathogenesis of MASLD, targeted drugs for MASLD have not yet been developed. However, the prevalence of MASLD is statistically higher in T2DM patients; consequently, anti-T2DM drugs such as sodium-glucose cotransporter2 inhibitors, biguanides, and thiazolidinediones are often employed for the treatment of MASLD in the clinical setting [146]. Noteworthily, insulin resistance plays a central role in obesity-related MASLD. It is reported that gremlin-1, a novel secreted insulin antagonist, facilitates MAFLD development according to in vivo and in vitro observation [147]. This effects were accompanied by elevated ER stress and inhibition of autophagy pathway [147]. Resveratrol effectively decreases the levels of fatty liver biomarkers and confers protection against MASLD in animal studies. Nonetheless, further studies are needed to determine its clinical applicability [148]. Breviscapine directly inhibits the TGF-β-activated kinase 1 (TAK1) and is deemed a possible candidate for the treatment of MASH [149]. In recent years, the effect of gut microbes on MASLD has been a research hotspot. Prebiotics and probiotics are considered reasonable options for the prevention and treatment of MASLD [150]. The gut microbiota participates in intestinal energy metabolism, and their implication in the pathogenesis of obesity and MASLD is being increasingly recognized. Similarly, intestinal hormone pathways are potential therapeutic targets for the management of MASLD [151]. Newly identified targeted drugs like PPAR agonists, FXR agonists, and THR-β agonists are currently being investigated in clinical trials and are anticipated to be applied for the treatment of MASLD. To date, bariatric surgery can effectively control obesity and reduce prevalence of MASLD [152]. In addition, the possibility that bariatric surgery induces microbiome alterations and thus prevents MASLD cannot be excluded, a concept that holds promise for the development of novel therapeutic strategies [153].

## Strengths and limitations

This article focuses on the major pathogenic mechanisms of MASLD, including recent research advances in lipotoxicity and gastrointestinal microbiology, as well as possible therapeutic approaches and emerging targets for the treatment of MASLD. Moreover, the article offers a comprehensive overview of the latest research results on MASLD. Nevertheless, there are certain shortcomings that merit acknowledgment. Attributed to data limitations, the provision of clinical trial results on drugs for the treatment of MASLD is relatively lacking. In addition, the bidirectional relationship between pathogenesis and MASLD was not explored in this article. Finally, more research is necessitated to elucidate the intricate links between various mechanisms and the development of MASLD.

## Conclusion and outlook

**Fig. 4:**
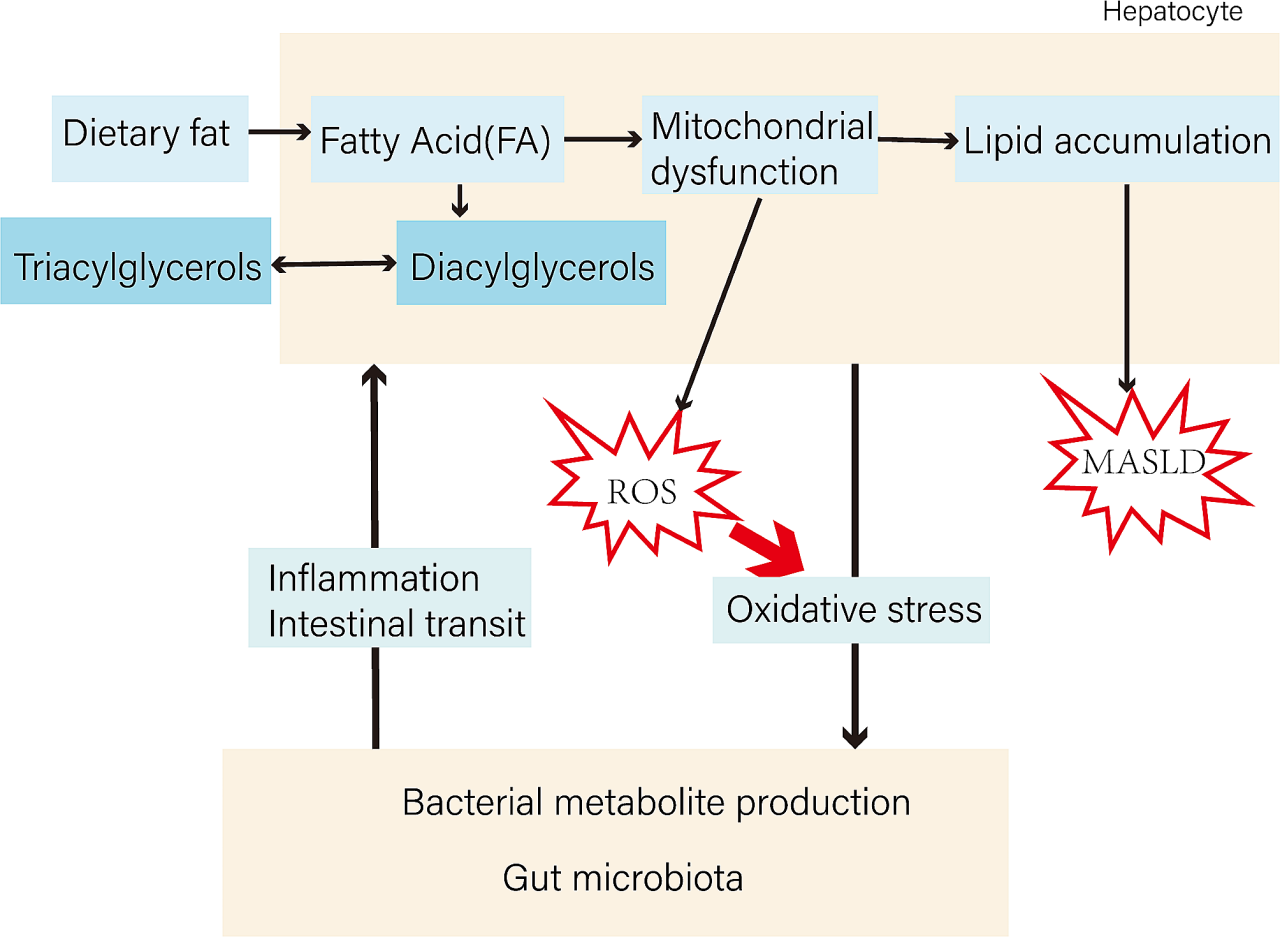
Schematic representation illustrating part of the pathogenesis of MASLD. Excessive dietary fat intake triggers multiple hepatic mechanisms, including mitochondrial dysfunction, which induces hepatic lipid accumulation, leading to MASLD. on the other hand, gut microbial dysbiosis is an essential component in the study of MASLD development, and the “liver-gut axis” has been proposed as a new target to drive the complex progression of MASLD.

The term MASLD encompasses a spectrum of liver diseases. Among them, MASH, which is featured as inflammation, steatosis, and cellular damage, can lead to liver fibrosis, cirrhosis, and hepatocellular carcinoma. Instead of the conventional two-hit doctrine, the multiple-hit theory is widely accepted, attributing the complex pathogenesis of MASLD to factors such as genetic susceptibility, epigenetic changes, signaling pathways related to hepatic lipid metabolism pathways, and dysbiosis of intestinal microflora. Excessive energy is stored in the body in the form of fatty acids, which are deposited in the liver through fat mobilization and DNL from substrates such as amino acids. Evidently, disturbances in fatty acid metabolism promote hepatic lipid deposition, and the resulting metabolite-induced lipotoxicity strikingly affects MASLD development(Fig. 4). Although MASLD has become a global health concern, pharmacological interventions for its treatment are in the developmental stage. So far, lifestyle interventions remain fundamental to the prevention and treatment of MASLD, with antidiabetic drugs as well as lipid-lowering drugs playing a complementary role. Research into the dysfunction of metabolism remains a key focus for pharmaceutical development, and the influence of gut microbes on MASLD is of interest as a potential therapeutic strategy (Fig. 4). Considering the global prevalence of MASLD and the relatively young age of disease onset, healthcare providers should be more vigilant in timely diagnosing the disease. Likewise, regular medical check-ups are essential for early disease diagnosis and prevention prior to the development of complications.

## Data Availability

No datasets were generated or analysed during the current study.

## Abbreviations

ACC Acetyl: CoA carboxylase
ADH: Alcohol dehydrogenase
AG: Alanyl-glutamine
APLN: Apelin
APOH: Apolipoprotein H
ATF6: Activating transcription factor 6
Bax: Bcl2-associated x
Bim: Bcl-2 interacting mediator
CA: Chicory acid
cGPX4: canonical-GPX4
CHOP: C/EBP-homologous protein
CHOs: Carbohydrates
ctsb: cathepsin B
DNL: De novo lipogenesis
eIF2α: eukaryotic initiation factor-2α
ER: Endoplasmic reticulum
FAO: Fatty acid oxidation
FAs: Fatty acids
FAS: Fatty acid synthase
FATPs: Fatty acid transport proteins
FFA: Free fatty acid
FFAR2: Free fatty acid receptor 2
FOs: Fish oils
GM: Gut microbes
GPX4: Glutathione peroxidase 4
GSH: Glutathione
HCC: Hepatocellular carcinoma
IF: Intermittent fasting
iGPX4: Inducible-GPX4
IRE1: Immunoglobulin-regulated enhancer 1
JNK: c-Jun N-terminal kinase
JNK: c-Jun N-terminal kinase
LJ2a: 6-methyl-tetrahydro-isoquinoline
LJ3a: 3-(S)-neopentyl proline
LPT1: Liproxstatin-1
MASH: Metabolic dysfunction-associated steatohepatitis
MASLD: Metabolic dysfunction-associated steatotic liver disease
MCD: Methionine-choline deficient
MD: Mediterranean dietary
NAFL: Non-alcoholic fatty liver
NAFLD: Nonalcoholic fatty liver disease
NASH: Non-alcoholic steatohepatitis
ND: Normal diet
Nrf2: Nuclear erythroid-related factor 2
OA: Oleic acid
PA: Palmitic acid
PERK: protein kinase RNA-like endoplasmic reticulum kinase
PUMA: P53-up-regulated modulator of apoptosis
SCFAs: Short-chain fatty acids
SFA: Saturated fatty acid
SFL: Simple fatty liver
TFR1: Transferrin receptor 1
TG: Triglyceride
TM6SF2: Transmembrane 6 superfamily member 2
TRIM59: Tripartite motif-containing
Tβ4: Thymosin beta 4
UPR: Unfolded protein response
VDAC: Voltage-dependent anion channel
XBP1: X-box binding protein 1
ZAF: Zeaxanthin
